# Podoplanin: Its roles and functions in neurological diseases and brain cancers

**DOI:** 10.3389/fphar.2022.964973

**Published:** 2022-09-13

**Authors:** Yi Wang, Dan Peng, Yaqian Huang, Yongjun Cao, Hui Li, Xia Zhang

**Affiliations:** ^1^ Department of Neurology, The Second Affiliated Hospital of Soochow University and Clinical Research Center of Neurological Disease, Suzhou, China; ^2^ Department of Cardiology, The Second Affiliated Hospital of Soochow University, Suzhou, China

**Keywords:** podoplanin, Clec-2, thrombosis, inflammation, angiogenesis, immune response

## Abstract

Podoplanin is a small mucin-like glycoprotein involved in several physiological and pathological processes in the brain including development, angiogenesis, tumors, ischemic stroke and other neurological disorders. Podoplanin expression is upregulated in different cell types including choroid plexus epithelial cells, glial cells, as well as periphery infiltrated immune cells during brain development and neurological disorders. As a transmembrane protein, podoplanin interacts with other molecules in the same or neighboring cells. In the past, a lot of studies reported a pleiotropic role of podoplanin in the modulation of thrombosis, inflammation, lymphangiogenesis, angiogenesis, immune surveillance, epithelial mesenchymal transition, as well as extracellular matrix remodeling in periphery, which have been well summarized and discussed. Recently, mounting evidence demonstrates the distribution and function of this molecule in brain development and neurological disorders. In this review, we summarize the research progresses in understanding the roles and mechanisms of podoplanin in the development and disorders of the nervous system. The challenges of podoplanin-targeted approaches for disease prognosis and preventions are also discussed.

## 1 Introduction

Neurological disorders constitute a major cause of disability and death, responsible for 16.8% total deaths worldwide as described by the Global Burden of disease Study 2015. Neurological disease-associated deaths increased 36% worldwide during the past 25 years (Gunata et al., 2020). Studies demonstrate that vasculopathy, inflammation and immune abnormality play important roles in the onset and progression of neurological diseases. However, the exact cellular and molecular mechanisms remain elusive, deserving further investigation.

Podoplanin (Pdpn), initially named due to its expression in renal podocytes), also known as PA2.26, gp36, T1α and aggrus, is a small sialomucin-like type I transmembrane glycoprotein (Toyoshima et al., 1995; Breiteneder-Geleff et al., 1997; Gandarillas et al., 1997; Ma et al., 1998; Zimmer et al., 1999). It is extensively expressed in different tissues and cells, including lymphatic endothelial cells, type I alveolar cells, osteocytes, choroid plexus epithelial cells, glial cells, and stromal reticular cells in lymphoid organs, participating in a plethora of processes such as organ development, thrombosis, lymphangiogenesis, angiogenesis and inflammation (Wetterwald et al., 1996; Breiteneder-Geleff et al., 1999; Scholl et al., 1999; Kato et al., 2008). In recent years, increasing studies reveal a role of Pdpn in the nervous system in healthy and diseased conditions (Table 1). Here we review and discuss the current knowledge on the roles and mechanisms of Pdpn in the nervous system development and disease progression, which may provide a potential target for the prognosis and/or intervention of neurological disorders.

**TABLE 1:** PDPN in the nervous system.

Diseases	Species	Trend	Outcomes	Potential Molecules	References
Brain development	Mouse	↑	the integrity of the developing vasculature	CLEC-2, NGF, ERM?	Lowe et al. (2015); Cicvaric et al. (2016)
			Neuritic outgrowth, synaptic plasticity of hippocampus-dependent		
Hemorrhagic stroke	Mouse	↑	the integrity of the developing vasculature	CLEC-2,T-synthase	Lowe et al. (2015); Hoover et al. (2021); Xia et al. (2004); Finney et al. (2012)
			prevent hemorrhage		
			correctly form blood-brain-barrier		
Ischemic stroke	Mouse	↑	platelet activation	CLEC-2, NLRP3?	Suzuki-Inoue (2017); Meng et al. (2021); Furukoji et al. (2019)
			thrombosis		
			ischemic-reperfusion injury		
			regulate inflammatory cytokines		
Brain tumors	Human	↑	high risk of death	CLEC-2, CD9, CD44, ERM, HSPA9,CCL21	Mishima et al. (2006); Ernst et al. (2009)
			cancer invasion and metastasis		
			platelet activation		
			thrombosis		
TBI	Mouse	↑	neuroprotection	CLEC-2	Guo et al. (2019)
			cerebral edema improved		
			regulation of inflammatory response		
Multiple sclerosis	Human	↑	regulation of inflammatory response	CLEC-2,Th17,IL-17	Miyamoto et al. (2013); Zhu et al. (2021); Noack et al. (2016b)
			prevent tissue damage		
Myasthenia gravis	Human	↑	regulation of inflammatory response	Th17	Agrawal et al. (2015)
			contribute to homeostasis		

## 2 Podoplanin structure, protein partners and cellular expression

**FIGURE 1:**
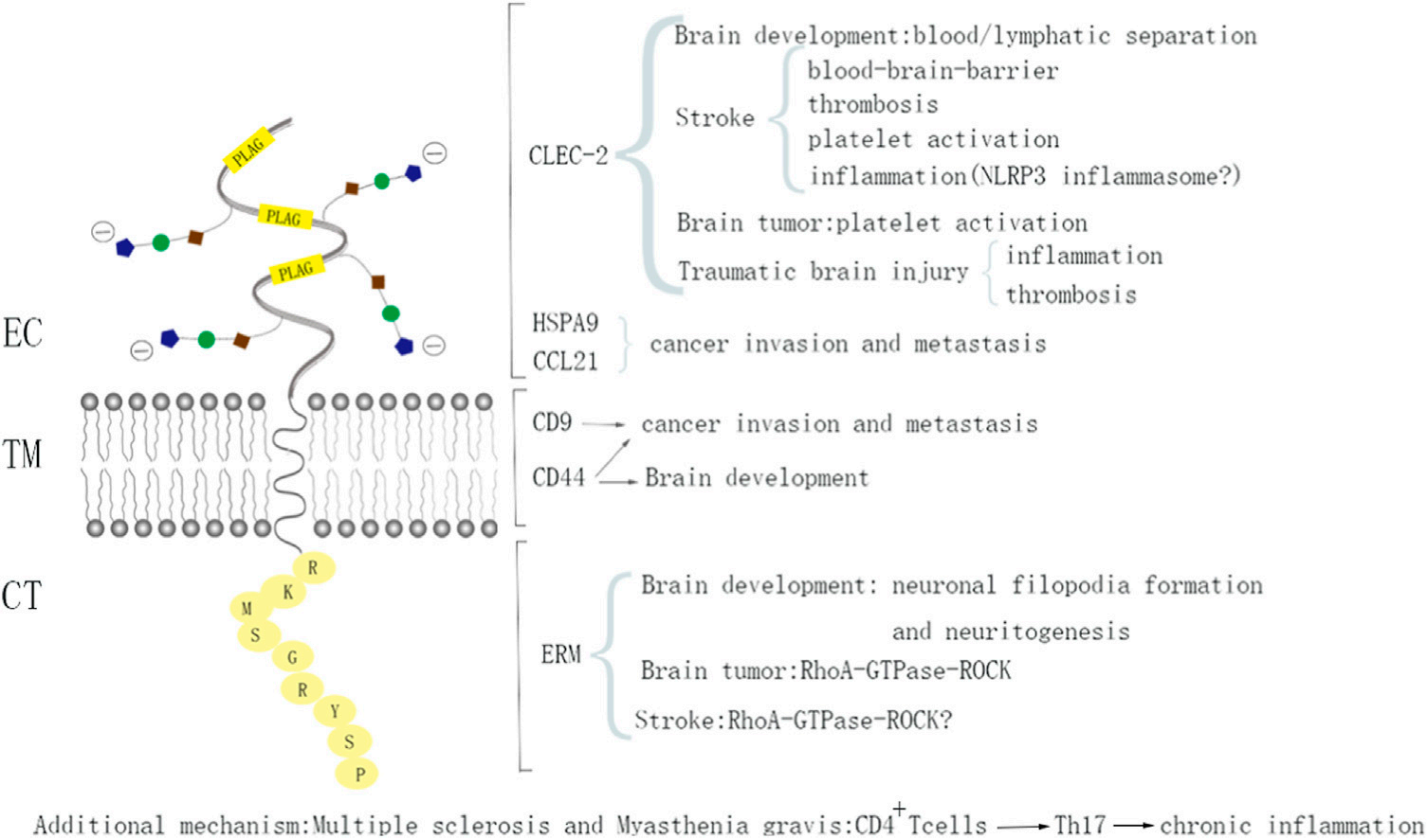
The structure of podoplanin and its functions with interacting proteins. Schematic representation of the molecular structure of podoplanin showing that it has a heavily glycosylated extracellular domain, a single transmembrane domain, and a short 9-amino acid cytoplasm. The ligands and biological processes during which the identified molecules interacting with podoplanin are presented. EC, ectodomain; TM, transmembrane region; CT, cytosolic domain.

Pdpn is composed of an extracellular domain with about 130 amino acids, a transmembrane domain with approximately 25 amino acids, and a short intracellular domain of approximately 10 amino acids (Martín-Villar et al., 2005). Due to the lack of enzymatic motifs, it exerts its function mainly through protein-protein interactions. Proteins bound to the extracellular domain of Pdpn include C-type lectin-like receptor-2 (CLEC-2), galectin-8, heat-shock protein A9 (HSPA9), and CCL21 (Tsuneki et al., 2013; Chen et al., 2016; Suzuki-Inoue et al., 2017; Tejchman et al., 2017). Notably, Pdpn is the only known ligand of CLEC-2. Under pathological conditions or during organ development, the interaction between Pdpn and CLEC-2 from platelets or hematopoietic cells results in platelet aggregation/activation, thrombosis, lymphatic vessel development, and cancer invasion and metastasis. The binding of Pdpn with galectin-8 supports the connection between the lymphatic endothelium and surrounding extracellular matrix. The HSPA9-Pdpn or CCL21-Ppdn interaction may contribute to cancer invasion. The transmembrane domain of podoplanin is known to bind to CD9 and CD44, which plays a role in cancer progression and lymph node expansion during adaptive immune activation (Nakazawa et al., 2008; Martín-Villar et al., 2010). Additionally, the intracellular domain of Pdpn can be bound by ezrin/radixin/moesin (ERM) proteins, leading to RhoA protein activation and epithelial-mesenchymal transition (EMT) in cancer cells (Krishnan et al., 2013; Krishnan et al., 2015; Suzuki-Inoue et al., 2017) (Figure 1).

Pdpn is expressed in the developmental and adult mammalian brain. In the early stage of embro development, Pdpn is widely expressed on neuro-epithelium along with the intermediate filament protein nestin throughout the neural tube. It interacts with CLEC-2 on platelets, mediating platelet adhesion, aggregation, and secretion, which is essential for the maturation and integrity of the developing cerebral vasculature (Lowe et al., 2015). By E14.5, Pdpn expression is localized to the ependymal lining of the ventricle wall and to the choroid plexus. Immunohistochemical studies showed that in the mouse brain, choroid plexus and ependyma were podoplanin-positive, and there were brain parenchymal cells expressing both podoplanin and the astrocyte marker GFAP in the cerebral cortex, hippocampus, thalamus, and fornix (Kaji et al., 2012; Tomooka et al., 2013). In addition, Pdpn is abundantly expressed in nestin-positive neural progenitor cells and in neurons of hippocampal dentate gyrus (DG) in the adult brain (Cicvaric et al., 2020). The distribution and functional studies from *Pdpn-deficient* mice suggest a critical role of Pdpn in the development and normal function of the brain.

## 3 Brain development

Pdpn is expressed in lymphatic endothelial cells, and its interaction with platelet CLEC-2 is essential for lymph/blood vessel separation at the embryonic stage (Suzuki-Inoue et al., 2011). Besides, Pdpn plays a role in angiogenesis during development. Lowe et al. reported that cerebral vessels were tortuous and abnormally patterned at E10.5, preceding the formation of large brain hemorrhages in *Pdpn* deficient mice. Pdpn on the neuroepithelium surrounding cerebral vessels was proposed to bind to CLEC-2 on leaking platelets, resulting in platelet aggregation and secretion (Lowe et al., 2015). A recent study demonstrated that Pdpn was temporarily expressed in neural tissue during midgestation and that loss of Pdpn resulted in overwhelmed activation of embryonic megakaryocytes, vascular defects and spontaneous hemorrhage in *Pdpn-deficien*t mouse embryos (Hoover et al., 2021).

Pdpn is also involved in neurite outgrowth, synaptic plasticity, and hippocampus-dependent memory functions as well. Pdpn may have a sub-region specific activity on hippocampal synaptic plasticity, since a relevant cross-talk between Pdpn and nerve growth factor (NGF)/TrkA signaling pathway exists, which is a key regulator of hippocampal synaptic plasticity and memory. Deletion of *Pdpn* impaired hippocampus-dependent spatial learning and memory without affecting amygdala-dependent cued fear conditioning (Cicvaric et al., 2016). The *in vitro* study showed that neuronal overexpression of Pdpn promoted synaptic activity and neuritic outgrowth whereas *Pdpn-deficient* neurons exhibited stunted outgrowth and lower levels of p-Ezrin, TrkA and CREB in response to NGF stimulation. Surface Plasmon Resonance data identify a physical interaction between Pdpn and NGF. Therefore, Pdpn may serve as a key molecule linking NGF/TrkA and the ERM protein family, and contribute to neuronal filopodia formation and neuritogenesis in the mammalian nervous system. This still warrants further investigation. Remarkably, Ppdn is predominantly expressed in proliferative nestin-positive adult neural progenitor cells and neurons in the hippocampal DG, a region related to neurogenesis. Additionally, Pdpn plays a role in the fine-tuning of hippocampal neurogenesis. *Pdpn* deletion enhanced the proliferative capacity of DG neural progenitor cells and diminished survival of differentiated neuronal cells *in vitro*, which was rescued by NGF treatment (Cicvaric et al., 2020). The underlying mechanisms remain to be elucidated, possibly due to its crosstalk with some functional interacting partners such as RhoA, CDC42, CD44, NGF, and especially Ezrin. NGF is a neurotrophin implicated in regulating hippocampus-mediated memory storage in Alzheimer’s disease (Iulita et al., 2017). The crosstalk between Pdpn and NGF may indicate a potential role of Pdpn in the pathogenesis of Alzheimer’s disease. Pdpn acts as one of the bidirectional regulators of memory-related synaptic potentiation or depression *in vivo*. Mice with *Pdpn* disruption showed anxiety-like behaviors (Cicvaric et al., 2020).

## 4 Relevance of podoplanin to diseases

### 4.1 Nonmalignant neurological disorders

#### 4.1.1 Stroke

##### 4.1.1.1 Hemorrahagic stroke

Pdpn plays an important role in the development and integrity maintenance of cerebrovascular tissue. The blood-brain-barrier (BBB) provides a barrier between blood and interstitial fluid, which is composed of neurovascular units (NVUs). And the loss of T-synthase, a key glucosyltransferase responsible for glycosylation of the extracellular domain of Pdpn, causes the formation of a disorganized microvasculature network with a defective recruitment of extracellular matrix (ECM) and pericytes, leading to BBB disruption and even cerebral hemorrhage (Xia et al., 2004). Moreover, the choroid plexus which is responsible for cerebrospinal fluid (CSF) secretion, expresses Pdpn early from embryonic development till adult stage. These Pdpn-expressing epithelial cells, which is distinct from the endothelium of BBB, form a barrier between blood and CSF. Finney BA et al. proposed that the platelet interaction with Pdpn-expressing cells in the choroid plexus may be essential for correct formation of the blood-CSF barrier. In addition, the interaction between Pdpn and platelet CLEC-2 induced platelets activation during fragile neovacularization (Finney et al., 2012). Platelet granule contents recruit pericytes, which in turn produce extracellular matrix to guide the maturation and integrity of the developing vasculature and prevent hemorrhage attack. Consistently, Lowe et al. observed large hemorrhages in *Pdpn*-deficient mice (Lowe et al., 2015). The formation of aneurysm and spontaneous hemorrhage, especially in the lower diencephalon during midgestation, was also reported in the developing brain of mice lacking *Pdpn* (Hoover et al., 2021). However, in mature brain, the role of Pdpn in hemorrhagic stroke is still unclear, which needs further exploration.

##### 4.1.1.2 Ischemic stroke

Thromboinflammation, a process related to the activation of both platelets and immune cells is increasingly recognized to participate in ischemic stroke (De Meyer et al., 2016). The interaction between Pdpn on activated macrophages and platelet CLEC-2 has been proven to play a role in thromboinflammation (Suzuki-Inoue, 2017). Our previous study revealed the prognostic significance of plasma CLEC-2 levels in acute ischemic stroke, and demonstrated that higher plasma CLEC-2 levels were associated with more progression and worse prognosis at 90 days after the onset of stroke and higher incidence of death and vascular events during 1 year of following-up (Zhang X. et al., 2018; Wu et al., 2019). We further reported anti-Pdpn treatment with its blocking antibody was beneficial against ischemic-reperfusion injury and reduced the yield of IL-18 and IL-1β in middle cerebral artery occlusion (MCAO) mice. The data indicate a role of the CLEC-2/Pdpn axis in the regulation of inflammation and ischemic stroke via modulating NLRP3 inflammasome (Meng et al., 2021). Additionally, Kolar K et al. observed an upregulated Pdpn expression in reactive astrocytes in the ischemic model and proposed this may be compensatory machinery to ischemic brain injury (Kolar et al., 2015). However, the cellular and molecular mechanisms that underlie the role of Pdpn in ischemic stroke is still unclear. Different types of nerve cells including neurons, microglia and astrocytes may be involved. And cell-to-cell communication cannot be ignored. Both vascular and neurovascular interactions may contribute to the pathophysiological process of ischemic stroke. Moreover, the cytosolic domain of Pdpn interacts with the ERM (ezrin, radixin, moesin) protein family, which is critical for small Rho GTPases. RhoA/ROCK signaling pathway in astrocytes is demonstrated to be essential for neurogenesis and angiogenesis after cerebral ischemia (van der Meel et al., 2011; Washida et al., 2011; Christie et al., 2013). Therefore, the crosstalk between Pdpn and ERM protein family in astrocytes during ischemic stroke deserves to be further explored.

#### 4.1.2 Atherosclerosis

Atherosclerosis is an inflammation-related vascular disorder caused by intima lipid accumulation and endothelial cell dysfunction. Thrombus formation on disrupted atherosclerotic lesion often leads to cardiovascular accidents and even cerebral ischemic stroke. Hatakeyama K et al. found that Pdpn expression in smooth muscle cells and macrophages increased with atherosclerotic progression in abdominal aortas obtained from 31 autopsy cases (Hatakeyama et al., 2012). Moreover, overexpression of *Pdpn* in endothelial cells induced endothelial detachment and thrombus growth in rat carotid artery, similar to the plaque erosion in human. The researchers found vascular endothelial growth factor (VEGF)-A derived from superficial smooth muscle cells (SMC) affected endothelial Pdpn expression and thrombus formation in advanced atherosclerotic lesions. Platelet aggregation was also enhanced by VEGF-A *via* modulating Pdpn expression and its interaction with CLEC-2 (Furukoji et al., 2019). These results remind us that in different phases of atherosclerosis, Pdpn expression is altered in different types of cells with distinct roles, which needs further exploration. On the other side, Pdpn was detected in the interior of advanced atherosclerosis plaque rather than on its surface, which blocked its access to CLEC-2, making the role of internal Pdpn controversial. Additionally, Pdpn may play a role in triggering plaque rupture (Hatakeyama et al., 2012). Pdpn is detected in stromal myofibroblasts, which might promote cell migration and invasion. This implicates that Pdpn distribution in atherosclerotic lesions is associated with vascular remodeling and disease progression (Kawase et al., 2008; Hoshino et al., 2011). Interestingly, pro-inflammatory cytokines were found to enhance Pdpn expression in stromal and endothelial cells, further supporting a correlation between inflammation and podoplanin functions in atherosclerotic plaques (Gröger et al., 2004; Suzuki et al., 2008). In a disturbed blood flow (d-flow) model, monocyte Pdpn was upregulated by d-flow, and myeloid-specific *Pdpn* deletion mitigated the subendothelial accumulation of platelets and monocytes/macrophages (Tang et al., 2021). All these suggest that Pdpn may contribute to the atherosclerosis development in both CLEC-2-dependent and independent manners. Moreover, Pdpn may be involved in atherosclerotic lesions via regulating functions and regeneration of adventitial lymphatic vessels (Zhang Y. et al., 2018; Drosos et al., 2019).

#### 4.1.3 Traumatic brain injury

Traumatic brain injury (TBI) represents a major cause of death and disability worldwide. It occurs in various forms ranging from mild alterations of consciousness to death, closely related to inflammation, hypercoagulation and apoptosis (Kelso and Genelman, 2014; Kaur and Sharma, 2018). Coagulopathy following TBI is a serious consequence due to early platelet activation. Additionally, exacerbated neuroinflammation following TBI causes secondary injury, which may persist for a long period. Guo M et al. showed a remarkably higher concentration of plasma CLEC-2 in TBI patients relative to healthy controls and found that incremental plasma CLEC-2 levels were intimately related to increased trauma severity and 30-days death (Guo et al., 2019). However, a mouse model of TBI showed a protective role of platelet CLEC-2 in the process of neuroinflammation after TBI (Gao et al., 2020). Recombinant platelet CLEC-2 administration altered the secretion of inflammatory cytokines, mitigated the brain edema, restored blood barrier integrity and improved the neurological function. Pdpn level was observed to rise early at 1 hour post TBI and lasted 7 days. This implies that CLEC-2 elevation might play a protective role following TBI. The association between elevated CLEC-2 levels and worse prognosis of TBI might reflect insufficient protection of CLEC-2 against traumatic injury, which varies greatly dependent on the region and degree of injury.

The function and underlying mechanism of Pdpn in TBI are not fully understood. In several inflammatory diseases, Pdpn was found to be co-localized to neurons, astrocytes and pericytes, implicating its multiple functions in neuroinflammatory processes (Song et al., 2014; Kolar et al., 2015; Noack et al., 2016a). Importantly, Pdpn expression is induced in ameboid and activated microglia after TBI. Pretreatment with rCLEC-2 transformed macrophage/microglia polarization *via* the mammalian target of rapamycin (mTOR) pathway, which was also correlated with Pdpn expression. Thus, CLEC-2/Pdpn axis may participate in TBI *via* the regulation of mTOR pathway (Chuang et al., 2015; Gao et al., 2020). Fei M et al. found the knockdown of *Pdpn* decreased the proportion of M1-like microglia and increased the M2-like microglia, accompanied by decreases in IL-1β and TNF-α and increases in IL-10 and TGF-β after TBI. Additionally, *Pdpn* knockdown impaired microglial mobility and phagocytosis and downregulated the expression of matrix metalloproteinases (MMP). These observations indicated an exacerbating effect of Pdpn on microglia-mediated neuroinflammation following TBI. Hence, targeting Pdpn may serve as a potential strategy for TBI treatment (Fei et al., 2020). Likewise, upregulation of Pdpn in reactive astrocytes was observed in a mouse model of a needle injury (Kolar et al., 2015). All these data demonstrate a role of reactive glial Pdpn in the processes of TBI; however, the cellular and molecular mechanisms remain unclear so far. Whether the role of CLEC-2/Pdpn axis in coagulation contributes to TBI is still unclear, and more researches are required.

#### 4.1.4 Multiple sclerosis and myasthenia gravis

Multiple sclerosis (MS) is an inflammatory demyelinating disease with neurodegeneration characterized by demyelinating plaques, neuronal and axonal loss, of which tertiary lymphoid organs (TLOs) with ectopic lymphoid follicles have been observed in CNS (Serafini et al., 2004; Magliozzi et al., 2007; Peters et al., 2011; Kipp et al., 2012; van Noort et al., 2012). TLOs participated in antigen presentation and contributed to the progression into chronic stage of this disease (Kuerten et al., 2012). The characteristic features of TLOs include compartmentalization of T and B cells, presence of lymphatic vessels, and high endothelial venules (HEVs) (Park and Choi, 2005; Manzo and Pitzalis, 2007; Manzo et al., 2010). Pdpn served as a novel lymphatic marker protein and was found to be highly expressed in perivascular inflammatory lesions, indicating signaling communications between inflamed brain vasculature and lymphatic proteins in MS. Besides, development of ectopic lymphoid follicles (eLF) was partly dependent on the cytokine interleukin 17 (IL-17) and the Th17 cell surface molecule Pdpn in experimental autoimmune encephalomyelitis (EAE), the animal model of MS (Jäger et al., 2009; Peters et al., 2011). Pdpn was identified as a specific cell-surface marker distinguishing IL-17-producing Th17 cells from other polarized T helper cells, such as Th1 and Th2 in mice (Peters et al., 2011; Miyamoto et al., 2013). Anti-Pdpn-treated mice had significantly reduced numbers of eLFs, indicating a critical role of Pdpn in eLFs formation in the brains of Th17 cell recipients. However, Peters A et al. observed no diminution of clinical progression in anti-Pdpn-treated Th17 cell recipients with the reduction of eLFs and they thought it was more likely that the clinical effects of eLF formation could not be observed in such a short acute disease model, which might be more apparent in a chronic disease model (Peters et al., 2011). Moreover, Nylander A et al. demonstrated Pdpn as a marker of a nonpathogenic Th17 cell subset and a negative regulator of pathogenic Th17 inflammation (Nylander et al., 2017). Similarly, Pdpn neutralization negatively regulated pathogenic inflammation by inhibiting the differentiation of Th17 cells in cardiac eLF formation in viral myocarditis (Zhu et al., 2021). In rheumatoid arthritis (RA) and psoriasis with similar pathogenic process as MS, Pdpn caused a high IL-17 secretion through the interaction between activated lymphocytes and mesenchymal cells (Noack et al., 2016b; Noack et al., 2016a).

Different subpopulations of Th17 cells express Pdpn at a distinct level. Unlike in mice, human pathogenic Th17 cells express less Pdpn (podoplanin-negative) than non-pathogenic Th17 cells (Pdpn-positive), which produce anti-inflammatory IL-10 instead of pro-inflammatory IL-17. In a pro-inflammatory circumstance, during which Th17 cells differentiated toward a pathogenic phenotype, Th17 cells exhibited reduced expression of Pdpn associated with increased IL-17 production, indicating an inhibitory effect of human Pdpn-positive Th17 cells on inflammation, rather than promoting it (Nylander et al., 2017). Also, the interaction of soluble CLEC-2 and Pdpn ameliorates the Th17 inflammatory response, which is restored by *Pdpn* silencing (Agrawal et al., 2015; Nylander et al., 2017). Consistently, enhanced Pdpn expression was also observed in Th17 cells in myasthenia gravis, and this was responsible for the loss of B-cell tolerance and probably ectopic germinal center (eGC) antibody secretion process (Villegas et al., 2019). Hence, Pdpn may serves as a negative regulator of Th17 inflammation.

### 4.2 Neoplastic conditions in brain

#### 4.2.1 Brain tumors

Pdpn is identified in several types of tumors in the central nervous system (CNS), such as ependymal tumors, choroid plexus papillomas, meningiomas, astrocytic tumors, medulloblastomas, and hemangioblastomas. The expression in astrocytic tumors seemed to be associated with pronounced fibrous properties or malignant phenotype, as shown by high-frequent expression in pilocytic astrocytomas and glioblastomas (Shibahara et al., 2006). Pdpn mRNA and protein expression were markedly higher in glioblastomas than those in anaplastic astrocytomas (Mishima et al., 2006). Strong expression of Pdpn in high-grade gliomas was reported and proposed as a potential indicator for malignant progression and poor prognosis in glioma patients (Mishima et al., 2006; Ernst et al., 2009; Peterziel et al., 2012). Pdpn expression is associated with malignant progression involving epithelial-mesenchymal transition, metastasis, and invasion (Wicki et al., 2006; Kunita et al., 2007; Kato et al., 2008). *Pdpn* overexpression promoted the migration of glioma cells but had little effect on cell growth (Sun et al., 2020). But, Eisemann T et al. pointed out that deletion of *Pdpn* in primary glioblastoma cells or cell lines does not affect tumor progression in a mouse xenograft model, regardless of an evident Pdpn upregulation and its association with higher aggressiveness. This implicates that Pdpn inactivation does not represent a promising option for glioblastoma therapy. However, it may still be clinically used as an auxiliary marker for tumor progression (Eisemann et al., 2019). Pdpn may play distinct roles in different types of brain tumors according to their fibrous properties or malignant phenotype. And the potential malignant function of Pdpn may be compensated by another yet unknown protein, or Pdpn is associated with but not functionally implicated in malignant features of brain tumors.

Notably, Pdpn expression in primary brain tumors induced platelet aggregation and increased the risk of venous thromboembolism via its interaction with CLEC-2 (Riedl et al., 2017). Nazari PMS et al. explored the intercorrelation between intratumoral Pdpn expression and the isocitrate dehydrogenase 1 (IDH 1) mutation, and their mutual impact on VTE development in brain tumors, and found the risk of VTE in patients with IDH1 wild-type tumors was strongly correlated to Pdpn expression levels (Mir Seyed Nazari et al., 2018). Thus, inhibition of Ppdn may become a potential anti-cancer approach at least for patients with wild-type IDH1 tumors. Novel recombinant anti-Pdpn immunotoxin therapy showed a delay of tumor growth and enhanced the survival in intracranial tumor models (Chandramohan et al., 2013). Another cancer-specific monoclone Ab (casmab) against human Pdpn was shown to react with Pdpn-expressing cancer cells but not normal cells, which is expected to be helpful for molecular targeting therapy against Pdpn-expressing cancers (Kato and Kaneko, 2014).

Several mechanisms that underlie the contribution of Pdpn to cancer have been proposed. On one hand, the interaction between cancer cells expressed Pdpn and platelet CLEC-2 activates platelets and thus facilitates hematogenous cancer metastasis and cancer-associated thrombosis (Suzuki-Inoue., 2019). Meanwhile, growth factors secreted from activated platelets promoted tumor formation and angiogenesis. On the other hand, the Pdpn binding to ERM proteins through a juxtamembrane cluster of basic amino acids within the cytosolic domain, triggered the activation of RhoA GTPase-ROCK signaling, which eventually caused ezrin protein remodeling and promoted tumor metastasis and invasion (Martín-Villar et al., 2015; Petropoulos et al., 2018). There is also evidence reporting a role of Pdpn in tumor angiogenesis (Grau et al., 2015; Li et al., 2017). The conditioned medium obtained from *Pdpn* overexpressing glioma cells strongly induced angiogenesis *in vitro* compared to the mock transfected cells (Grau et al., 2015).

## 5 Conclusion and future perspectives

The glycoprotein Pdpn is increasingly revealed to have different roles in brain development and several neurological diseases including stroke, multiple sclerosis, myasthenia gravis, brain tumors and traumatic brain injury. It functions via the modulations of platelet, microglia or astrocyte activation, cytoskeleton remodeling, as well as Th17-mediated immune reactivity. Especially in brain I/R injury, Pdpn has been demonstrated to play a crucial role, however without clear mechanisms. An upregulated Pdpn expression in reactive astrocytes in the ischemic model and the possible crosstalk among Pdpn, ERM protein family and RhoA/ROCK signaling pathway in astrocytes indicated that Pdpn may contribute to brain I/R injury through the interaction between astrocytes and other cells in the brain such as neurons or vascular endothelial cells. In addition, neurovascular machinery due to the interaction between astrocytes or macrophages-derived Pdpn and platelet CLEC-2 may also be involved, which needs to be further explored. In general, rapid progresses have been made during the last decade; however, many issues remain to be elucidated further. For instance, cell/stage-specific effects of podoplanin and the relevance of anti-PDPN therapy on neurological development and disorders still warrant further investigations from bench to clinical translation.
